# Blockchain-Facilitated Cybersecurity for Ubiquitous Internet of Things with Space–Air–Ground Integrated Networks: A Survey

**DOI:** 10.3390/s25020383

**Published:** 2025-01-10

**Authors:** Wenbing Zhao, Shunkun Yang, Xiong Luo

**Affiliations:** 1 Department of Electrical and Computer Engineering, Cleveland State University, Cleveland, OH 44115, USA; 2 School of Reliability and Systems Engineering, Beihang University, 37 Xueyuan Road, Beijing 100191, China; ysk@buaa.edu.cn; 3 School of Computer and Communication Engineering, University of Science and Technology Beijing, Beijing 100083, China; xluo@ustb.edu.cn

**Keywords:** blockchain, space–air–ground integrated networks (SAGIN), dynamic spectrum management, mobility management, Internet of Things (IoT), smart contract, decentralized consensus, data immutability, security, trust, hyperledger, practical Byzantine fault tolerance (PBFT)

## Abstract

This article presents a systematic review on blockchain-facilitated cybersecurity solutions for Internet of Things (IoT) devices in space–air–ground integrated networks (SAGIN). First, we identify the objectives and the context of the blockchain-based solutions for SAGIN. Although, typically, the blockchain is primarily used to enhance the trustworthiness of some systems or operations, it is necessary to document exactly in what context the blockchain is used that is specific to the IoT and SAGIN. Second, we investigate how blockchain technology is used to achieve the objectives. Again, we want to report the technical details on how blockchain is used in this specific field instead of general discussion. Third, we provide a critique on the technical correctness of the blockchain-based solutions. As we elaborate in this article, there are serious technical issues in the proposed solutions. The most pervasive assumption made in many blockchain-based solutions is that higher-level trustworthiness can be achieved by using any form of blockchain. Fourth, we provide a guideline on when blockchain technology could be useful for IoT and SAGIN and what types of blockchain could be useful to enhance the security of ubiquitous IoT in SAGIN.

## 1. Introduction

The space–air–ground integrated network (SAGIN) is an emerging communication technology that is currently under rapid development [1,2,3]. It is generally regarded as a core component in next-generation 6G technology [4,5] because SAGIN implements the vision of 6G, which is to provide ubiquitous high-bandwidth connectivity to all entities on planet Earth, particularly the Internet of Things (IoT) [6]. Current data communication is predominately limited to terrestrial applications, i.e., at the surface of the Earth. Furthermore, terrestrial connectivity is usually available only in relatively densely populated regions [7]. Satellites could enable global connectivity. With the success of StarLink [8], satellite-based connectivity is becoming increasingly affordable. In addition to these two segments of networks (i.e., ground and space), unmanned aerial vehicles, airships, and balloons have been proposed to provide enhanced connectivity and to meet the network traffic demand as an aerial segment [9]. Because SAGIN consists of highly heterogeneous devices and several modalities of communication, it is essential for SAGIN to support optimal dynamic spectrum management, allocate computational resources, and ensure proper security for all its operations [1,2,3].

The Internet of Things (IoT) is closely related to SAGIN and 6G. As for many terms in the field of information, computers, and communication, there is no universally accepted definition for IoT despite being heavily used in the literature [10,11,12,13]. As the name suggests, the IoT is first and foremost a type of Internet that consists of “things”. Perspectives on how to interpret the “things” also differ greatly. On the one hand, it could mean embedded devices that are equipped with a variety of sensors with wireless connectivity. On the other hand, it could mean any connected entities, including entities as large as vehicles. For the latter case, we have seen phrases such as “Internet of Everything” [14] and “Internet of Vehicle” [15] in some literature. In the early days, the “things” are not really directly connected to the Internet; rather, they are connected to a computing device via Bluetooth or some other short-range wireless communication techniques. In recent years, we have seen more and more “things” that are capable of connecting to the Internet via WiFi or cellular connections. In this sense, the IoT can be considered a version of the Internet that greatly enhances the traditional Internet with sensing capabilities [16]. In [16], the notion of a ubiquitous IoT was introduced; the term refers to a three-dimensional network, i.e., SAGIN. Indeed, one could argue that it is the presence of the IoT that drives the need for the development of three-dimensional connectivity with high bandwidth and low latency.

Blockchain technology [17] is a decentralized computing technology introduced as part of Bitcoin, which is the first cryptocurrency released in January 2009 [18]. The most prominent innovation of blockchain technology is decentralized consensus, i.e., proof of work. Unlike traditional distributed consensus [17], proof of work enables a large-scale open system to achieve decentralized consensus without the notation of membership and without voting among participating nodes [19,20]. In 2015, Ethereum made a major enhancement to the original blockchain technology by adding support for Turing-complete smart contracts [21], which would facilitate deterministic execution of Turing-complete programming code in a decentralized system. Blockchain technology is regarded as offering stronger security and trust due to its unique characteristics not seen in traditional systems, including decentralization, censorship resistance, data immutability, and transparency [22]. As such, it is not surprising that blockchain has been proposed to enhance security and facilitate secure cooperation and collaboration in virtual all industry sectors [23,24,25,26,27,28,29,30], including SAGIN-based systems.

Despite the fact that SAGIN is still an emerging field, it has been reviewed numerous times (for example, [3]). These reviews have usually adopted a broad interpretation [3,13] of SAGIN and include studies that focus on any of the three segments, i.e., terrestrial, aerial, or space. Considering that terrestrial communication has been extensively studied over the last several decades, such studies could obscure the key challenges in offering integrated communication across ground, aerial, and space segments. Two reviews considered the roles played by the blockchain in SAGIN [3,13]. The review by Wang et al. [3] suffers from exactly this problem. The studies considered by that review included virtually all types of IoT applications. As such, that review may not inform how blockchain can be used to address the specific challenges in SAGIN. Another comprehensive review outlined how blockchain technology could be used in SAGIN [13]. Unfortunately, the relevant content in that review lacks technical details because the use of blockchain in SAGIN was not the focus of the review. Therefore, we argue that there is a need to systematically review studies regarding how blockchain technology has been used to address the cybersecurity issues in SAGIN and identify new research opportunities.

This comprehensive review is guided by the following research questions. First, what SAGIN operations are enhanced by the use of blockchain technology? Second, how is blockchain technology used in solutions to enhance SAGIN operations? Third, are the proposed blockchain-based solutions valid for the intended purposes and technically sound?

The first two questions are obvious. The first question is necessary so that the context of the blockchain application is clearly defined. The investigation for the second question has value because more knowledge and insight could be gained by examining the technical details on how blockchain technology is used to enhance the security of SAGIN operations. The third question might appear to be odd because peer-reviewed publications, in general, should have been validated for their technical merit and, particularly, should not contain serious or apparent technical mistakes. Unfortunately, this is not the case for the application of blockchain technology. The technical innovation of blockchain technology is deep and profound. A person without proper training in distributed algorithms (particularly distributed consensus) may not truly appreciate the innovations brought about by blockchain technology [17]. Furthermore, traditional distributed consensus algorithms are highly sophisticated and can be easily misunderstood [31]. Due to the popularity of blockchain technology, researchers who have barely any training in distributed algorithms in a variety of disciplines have rushed to incorporate blockchain in their research. Likewise, many researchers who have served as peer reviewers also do not have adequate training in distributed algorithms. After all, the field of distributed algorithms is a niche discipline in computer science, and a very small fraction of professionals are doing research in this field.

Given the issues identified with respect to the findings in response to the research questions, we aim to formulate a guideline on the adoption of blockchain technology in SAGIN operations and applications. We recognize that in the context of SAGIN operations and applications, decentralization and data immutability are not necessarily the most essential requirements. Quite often, what is needed is a dependable distributed system with specific functionalities. In this case, a custom private blockchain would be a good fit, provided that a sound distributed consensus algorithm is used. By providing a guideline, we hope to encourage the development of more practical blockchain-based solutions that could make SAGIN and its applications more useful and resilient to faults and cyber attacks.

The remainder of this article is organized as follows. Section 2 outlines the methodology used for literature collection. Section 3 provides a concise introduction to SAGIN. We intentionally omit an introduction to blockchain. Interested readers are referred to another article we authored [32] (Section 3) for this information. Section 4 reports our findings for the first research question regarding SAGIN operations and applications that have been enhanced by blockchain technology. Section 5 elaborates on the findings for our second research question on how blockchain technology is used in the proposed solutions. Section 6 presents the findings of the third research question, including the validation method and technical issues that we identified. Section 7 is centered around a guideline for adopting blockchain technology in SAGIN. Section 8 concludes this article.

## 2. Method of Literature Collection

**Figure 1:**
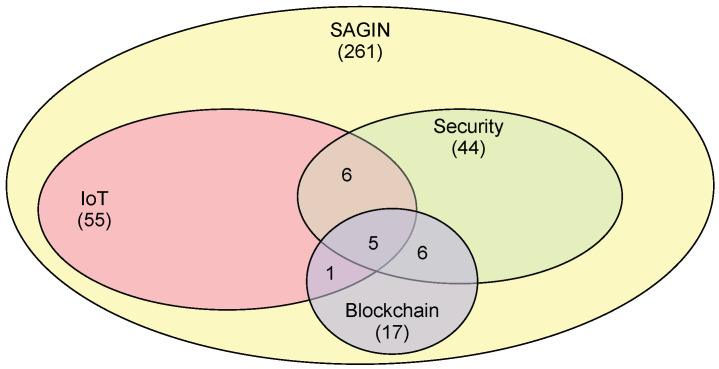
The search results with different sets of search terms.

The literature collection is based on the Web of Science core collection because this academic paper repository is the most reputable platform due to its high standard. We used the following search terms for our study: “SAGIN”, “SAGIN and IoT”, “SAGIN and blockchain”, “SAGIN and security”, “SAGIN and IoT and blockchain”, “SAGIN and security and blockchain”, and “SAGIN and IoT and blockchain and security”. The search outcome is reported in Table 1 in detail and is summarized graphically in Figure 1.

Although we do not plan to go over all studies returned by using the term “SAGIN”, we did go through the title information so that the total number of studies is accurate. Among the 261 entries returned, we identified 2 studies that have nothing to do with SAGIN. In one of the two studies, “sagin” refers to a newly discovered plant. In the other study, “Sagin” refers to the last name of a scientist. Hence, the number of studies actually relevant to “SAGIN” is 259. The returns obtained using other search terms are all relevant subject-matter-wise. Due to the focus on blockchain in this study, all 17 publications for “SAGIN and blockchain” were retrieved and examined. There are 11 overlaps between the entries returned by the search term “SAGIN and blockchain” and the search term “SAGIN and security”. The title and abstract of each of the remaining non-overlapping entries were examined, and full papers were retrieved only when needed. The reason for this secondary-level examination is to identify potential opportunities for adopting blockchain in SAGIN operations and SAGIN applications.

After the full papers for the 17 publications returned from the search term “SAGIN & blockchain” were retrieved and examined, we found that one paper only mentioned blockchain in the related work section and that blockchain was not considered in the study. Another paper (a review paper on digital twin edge networks) listed blockchain as one of the many enabling technologies, and SAGIN was mentioned as one of the applications. Hence, these two papers were excluded from our study. One of the studies that we included is a comprehensive survey [3].

## 3. Space–Air–Ground Integrated Networks

**Figure 2:**
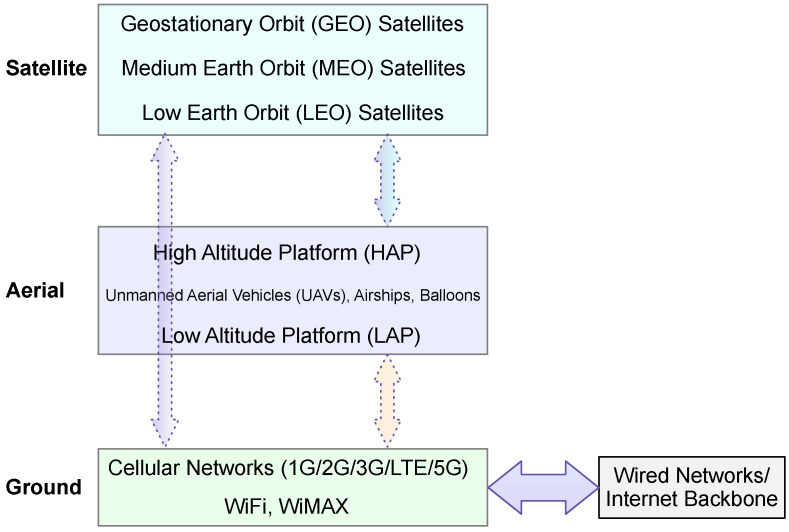
The architecture of the satellite–aerial–ground integrated network.

As shown in Figure 2, SAGIN reflects the vision for the next generation of wireless communication [2]. The core challenge of SAGIN is the integration of satellite, aerial, and terrestrial wireless communication. Although the focus of SAGIN is wireless communication, it does depend on the wired Internet backbone for global connectivity and data propagation. More specifically, the wired Internet backbone serves as the underlying infrastructure for high-capacity data exchange between different segments of the wireless network. Typically, the satellite and the aerial layers are set up to support IoT devices in the ground layer. However, there are use cases for satellite–aerial communication [2]. Within-segment networking is also possible in the satellite and aerial segments [2].

In Table 2, we highlight the key characteristics of SAGIN for our study, namely the best or worse case of signal propagation distance and one-way propagation delay. One-way propagation delay is estimated by dividing the propagation distance by the speed of light (using 30,000 km/s in our study). For the space segment, the best-case propagation delay is determined by the altitude of the satellites, which is 35,796 km for geostationary satellites (GEO) [2], 2000 km–35,786 km for medium Earth orbit (MEO) satellites [2], and 160 km–2000 km for low Earth orbit (LEO) satellites [2]. For the aerial segment, it is also interesting to see if the altitude has any substantial impact on the minimum propagation delay. In the aerial segment, typically, various unmanned aerial vehicles (UAVs), airships, and balloons are used at two different levels of altitude, referred to as high-altitude platforms (HAPs) and low-altitude platforms (LAPs) [2]. The range of altitudes for HAPs differs across studies. For example, the range given in [2] is 17 km–30 km, the range given in [33] is 17 km–25 km, and the range given in [34] is 17 km–22 km, corresponding to the altitude of the stratosphere, which has relatively mild wind and turbulence. The range of altitudes for LAPs is generally defined as below 10 km [2,35]. As can be seen in Table 2, the space–ground propagation delay could be significant for all but some low-altitude LEO satellites. The air–ground propagation delay is less than 0.1 ms; hence, it is negligible. Although ground-to-ground communication is obviously not limited by the altitude of transmission stations, the geodesic distance between a pair of transmission stations could be significant. Considering that the circumference of the Earth is 40,075 km, the worst-case propagation delay for ground-to-ground communication could be as large as 133.6 ms. That said, if the transmission stations are close to each other, the propagation delay is negligible. Due to the relatively low altitude of HAPs and LAPs, the propagation delay for space–air communication could also be significant. Transmission delay could be significant if the bandwidth is limited. Indeed, in the early development of communication satellites, the bandwidth was limited [36]. However, in recent years, bandwidth has typically exceeded Gbps [2], which has made the transmission delay negligible.

**Figure 3:**
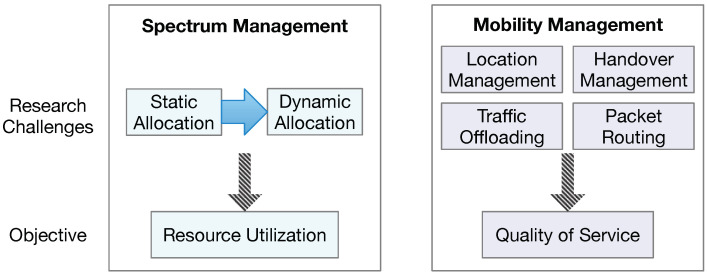
Key SAGIN challenges.

SAGIN faces highly complex research and development challenges. Here, we only go over two technical challenges that blockchain technology might play some positive role in addressing, i.e., spectrum allocation and mobility management, as shown in Figure 3. The goal of spectrum management is to maximize resource utilization. The goal of mobility management is ensure a high quality of service for users.

Spectrum allocation is essential for space–ground and space–air communication. Currently, the spectrum is allocated statically. What is needed in the future is to allocate the spectrum dynamically based on a number of factors, such as channel conditions and the users’ needs.

Unlike the wired Internet, SAGIN supports mobile users and also relies on communication entities possibly moving at high speeds, such as non-GEO satellites and UAVs. This requires carefully crafted mechanisms for mobility management to ensure a high quality of service for users in SAGIN. More specifically, mobility management is important to ensure a non-interrupted connection between two communicating mobile users. Mobility management consists of four major components:

- Location management is necessary for the network to track the locations of mobile users so that data packets can be routed and delivered properly. Mobile user equipment is required to register its location once it is moved to a new cell.
- Handover management refers to the quick transfer of an ongoing connection from the original connected cell to a new one so that the connection is not broken.
- Traffic offloading is a practice used to move traffic from one network to another network due to a variety of reasons, such as capability limitations. For example, when the ground segment becomes heavily congested, it is desirable to move some traffic to the aerial or space segment.
- Packet routing involves the possible need for data packets to traverse between a number of communication devices. The aim of packet routing is to determine the most optimal route for the packets.

## 4. RQ1: What SAGIN Operations Are Enhanced by the Use of Blockchain Technology?

**Figure 4:**
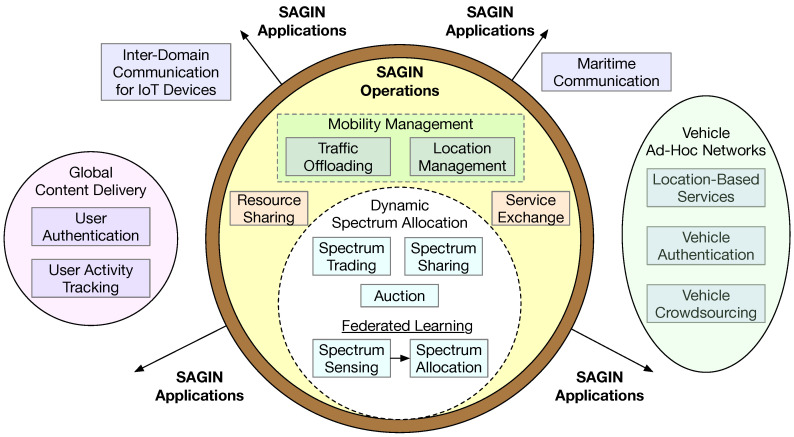
SAGIN operations and applications facilitated by blockchain technology.

The findings in response to this research question are summarized in Figure 4 and Table 3. Blockchain technology has been proposed to enhance the security of most of the SAGIN core operations that we highlighted in Figure 3. Particularly, blockchain has been incorporated as a key building block for solutions to achieve dynamic spectrum allocation [37,38,39]. Several approaches have been adopted for dynamic spectrum allocation. Spectrum owners could trade with each other [37]. Spectrum owners (i.e., primary users of the spectrum) could decide to share with secondary users via auction or some other schemes for a fee [38,39]. For optimal spectrum allocation, it is essential to dynamically detect when the spectrum is available and when the spectrum is congested, i.e., spectrum sensing for availability and interference [38,39]. For this, federated learning has been proposed to dynamically determine the available spectrum and, correspondingly, an optimal spectrum allocation scheme [38]. Subsequently, the optimal allocation scheme can be enforced via a smart contract [38]. In addition to dynamic spectrum management, traffic offloading [40] and location management [40] in mobility management have also been addressed by blockchain-based solutions.

Some studies have expanded the scope of spectrum management to resource sharing [41,42]. A spectrum is one form of resource (also referred to as a bandwidth resource) [42]. Other resources include energy (important for battery-powered equipment, such as UAVs and IoT devices) and time [42]. Computation may also be regarded as a form of resource [41], but computation could be treated as a form of service; correspondingly, service exchange has been proposed for integration with blockchain technology, providing a trusted trading environment [41].

Besides the blockchain-based solutions for key SAGIN operations, several studies have reported the use of blockchain technology in supporting SAGIN applications. As shown in Figure 4, vehicle ad hoc networks appear to be a popular domain where blockchain technology has been proposed to enhance security in location-based services [43], vehicle authentication [44,45], and vehicle crowdsourcing [46]. In conjunction with mobile edge computing and SAGIN, a blockchain-enabled solution was proposed for global content delivery [47]. Two other studies focused on using blockchain to enhance the security of maritime communication [48] and communication between IoT devices [49].

**Table 3:** SAGIN operations and applications facilitated by blockchain technology, explanation, rationale, and references.

SAGIN Operations and Applications	Further Explanation	Rationale for Using Blockchain	Reference(s)
Dynamic Spectrum Management	Auction-based dynamic spectrum allocation	To address threats to traditional auction-based solutions	[37]
Dynamic Spectrum Management	Federated learning and smart contracts for spectrum sensing and allocation	Automated cooperation with smart contracts	[38]
Dynamic Spectrum Management	Identifying interference-dense subnetworks, interference-based spectrum pricing, and joint optimization	Decentralization	[39]
Mobility management	Traffic offloading and location management	Trusted information sources	[40]
Resource sharing	Resources include bandwidth/spectrum, energy, time, and computation	Trusted resource trading platform	[41]
Resource sharing and service exchange	Sharing is enabled by machine learning and blockchain	Blockchain technology is used for data immutability and traceability	[42]
Secure communication	To secure communication between IoT devices in different domains	To ensure data immutability	[49]
Secure communication	For maritime communication with mobile edge computing, blockchain, and SAGIN	Enhanced security, authentication, and automation with smart contracts	[48]
Global content delivery	For user authentication and user activity tracking	To ensure tamper-proofing, unforgeability, and non-repudiation	[47]
Vehicle ad hoc networks	Location-based services	To maintain trusted records	[43]
Vehicle ad hoc networks	Vehicle identity authentication	To enhance security	[44,45]
Vehicle ad hoc networks	Vehicle crowdsourcing	To provide a decentralized, trustworthy operating platform	[46]
General security architecture for SAGIN	Ground–space resource scheduling, air–space authentication, air–space mobility management, ground–air mobility management, and content broadcast	To enhance security	[50]

In [37], the dynamic spectrum allocation problem was addressed via blockchain-facilitated secure spectrum sharing. More specifically, a Vickrey auction mechanism [51] with incentive is proposed to enable spectrum sharing among UAVs. The stated security benefit of using a blockchain-based solution is that the characteristics of blockchain (“decentralization, non forgery, non fabrication, non tampering, whole process traceability, collective verifying” [37], page 20516) could effectively address two common types of attacks: (1) malicious spectrum bidder and (2) unreliable trust authority (if a centralized and trusted authority were to be used) attacks. The UAVs would use satellites for communication. The UAVs must register with the registration authority, which consists of satellite Earth stations and gateway stations. The registration authority is regarded as a trusted agent.

In [38], a solution to the dynamic spectrum allocation problem was proposed. The solution relies on the integration of federated learning a smart contract. Federated learning [52] is used to efficiently carry out spectrum sensing and spectrum allocation. A smart contract is used to enforce the spectrum allocation scheme derived from federated learning.

In [39], a blockchain-based solution was proposed for spectrum management in SAGIN. The basic idea is to use blockchain-facilitated decentralized spectrum management to maximize effective spectrum sharing among the users. Besides using blockchain, the authors proposed algorithms for identifying interference-dense subnetworks, interference-based spectrum pricing, and joint optimization of non-terrestrial nodes based on location and transmission power.

In [40], a blockchain-facilitated traffic offloading solution was introduced. Blockchain is used to help secure the sharing of network topological and model information for traffic offloading. More specifically, two separate blockchain systems were proposed. One is referred to as the global topology chain, which, obviously, stores the SAGIN topology information. The other is referred to as the global model chain, which stores the model information needed for federated reinforcement learning. The latter is introduced to make optimal decisions regarding traffic offloading. The two blockchains ensure that all nodes see the same topological and model information (i.e., they serve as trusted information sources), despite the presence of malicious nodes in the SAGIN network.

In [41], the computational and bandwidth resource allocation problems in SAGIN were studied. In addition to blockchain, multiaccess edge computing is considered another enabling technology. The study assumed a system model where IoT devices are the primary users of SAGIN. The UAVs in the aerial segment and the LEO satellites in the space segment serve as the multiaccess edge computing servers. The objective of the study was to achieve minimum long-term energy consumption of all IoT devices while satisfy the task completion requirements assigned to the IoT devices. The tasks assigned to IoT devices may be offloaded to the edge servers (i.e., LEO satellites and UAVs). The blockchain is used to ensure that the task processing is trustworthy. The proposed blockchain would run on the UAVs, and LEO satellites would serve as the clients of the blockchain.

In [42], a resource-sharing and service exchange scheme was proposed. The scheme is powered by machine learning (for optimal decision making) and blockchain (to provide a trusted trading environment). Resource sharing and service exchange take place between two ground base stations. Resources that could be shared include spectrum, energy, and time. Services that could be exchanged include the relaying and transmission of packets and computing. Data immutability and traceability were cited as the reasons for using blockchain.

In [50], a blockchain-based architecture was proposed to enhance the security of SAGIN. The provided description is at a very high level without in-depth technical details. The paper claims that the architecture could facilitate many key operations relevant to SAGIN, including ground–space resource scheduling, air–space authentication, air–space mobility management, ground–air mobility management, and content broadcast.

In [49], blockchain was used to enhance secure communication for SAGIN-facilitated IoT applications. The paper assumed that LEOs are used to support inter-domain communication. The paper did not clearly define what a domain. For the structural diagram in the paper, it appears that the domain refers to a group of IoT devices for a specific purpose, such as smart medical, smart grid, and smart city applications. The stated goal of using blockchain technology is to ensure data immutability, among other things (such as user authentication and data sharing).

In [43], blockchain technology was used to address issues related to the application of SAGIN instead of core SAGIN operations. More specifically, blockchain was used to facilitate trusted location-based services in vehicle ad hoc networks. The paper argued that to provide ubiquitous connectivity to connected vehicles, SAGIN is necessary. The study proposed two custom blockchains; one blockchain is used to store all requests made by the vehicles, and the other blockchain is used to store certificates issued to the vehicles. All vehicles would be required to registered with a registration authority, and upon registration, a vehicle would be given a certificate. The road system is divided into multiple roadside units. The records stored in the request blockchain are used as the trusted basis for the credibility of roadside units and in case of dispute about registration with the registration authority.

Ref. [44] also focused on addressing the security issues in vehicle ad hoc networks. SAGIN is assumed to provide the necessary ubiquitous connectivity for the vehicles. A custom blockchain called hashchain was introduced to facilitate vehicle identity authentication in a “distributed and decentralized” ([44] 2nd page and 4th page) manner. The authors argued that the proposed scheme offers stronger security than the traditional vehicle authentication structure. The journal version of the study [45] reported an identical design.

In [46], blockchain technology was identified as one of the components of SAGIN-supported vehicular crowdsensing. The motivation for using blockchain was to provide a decentralized, trustworthy operating environment.

In [47], blockchain technology was used to support another application of SAGIN (with mobile edge caching), i.e., global content delivery to users. A custom blockchain was proposed to provide trusted authentication and activity tracing for the edge caching system. The rationale for using blockchain is that blockchain would ensure the following benefits: (1) tamper-proofing, (2) unforgeability, and (3) non-repudiation.

In [48], blockchain was identified as one of several enabling technologies (along with mobile edge computing and SAGIN) for maritime communication. The stated benefits of using blockchain for maritime communication include enhanced security and privacy, efficient authentication, and automation with smart contracts.

## 5. RQ2: How Is Blockchain Used in the Solutions for Enhancing SAGIN Operations?

While there are only two public blockchain systems that are large enough to offer some degree of data immutability (i.e., Bitcoin and Ethereum) [53], numerous alternative blockchains have been created, such as PeerCoin [54], Nxt [55], IOTA [56], and layer-2 blockchain solutions [57]. There are also open-source blockchain projects that are meant to be used as a private or consortium blockchains, such as Hyperledger [58]. That is why it is quite surprising that almost all blockchain solutions for SAGIN choose to use a custom blockchain instead of an existing blockchain. Nevertheless, we summarize the findings with respect to the second research question in Table 4.

The description of the blockchain-based solution for spectrum sharing proposed in [37] is at a very high level. The solution is referred to as lightweight because a non-traditional, decentralized consensus algorithm is proposed. The so-called lightweight algorithm is based on delegated proof of stake. Furthermore, spectrum sharing is enabled by an incentive-based auction mechanism. Unfortunately, the description of the mechanism is purely algorithmic. There is no elaboration regarding how to implement the proposed mechanism in a blockchain-based system (such as via one or more smart contracts). The proposed solution consists of a custom private blockchain system among the UAVs.

In [41], each task processing operation was encoded as a transaction to be submitted to the custom blockchain. The blockchain nodes run on the UAVs. In [41], the PBFT (stands for practical Byzantine fault tolerance [59]) algorithm was used for the nodes to reach consensus. The purpose of the blockchain is to ensure the trustworthiness of task processing.

In [38], a smart contract was proposed to enforce a dynamically determined spectrum allocation scheme among the secondary spectrum users.

The blockchain-based solution for secure spectrum management proposed in [39] consists of blockchains at three different levels. This design is intended to address the limited throughput issue of public blockchains. At the lowest level are the local blockchains. Each local blockchain supports spectrum sharing between the primary users and the secondary users (the latter pay a fee for spectrum access). At the second level are the regional blockchains, which are tasked with supporting spectrum trading and carrying out interference control. At the highest level is a single global blockchain, which is tasked with data synchronization and cross-chain transactions. The authors stated that the local blockchains and the regional blockchains would be public blockchains and the global blockchain would be a consortium blockchain.

In [42], a custom blockchain was proposed to establish a general-purpose trusted trading environment between two ground base stations. To achieve higher throughput, the authors proposed the use of a consensus algorithm based on the directed acyclic graph algorithm as proposed for Tangle (introduced by the IOTA blockchain [56]).

In [40], two custom blockchains were proposed; each stores different information necessary for traffic offloading. The base stations in the ground segment are selected to run as the blockchain nodes. This study also chose to use the PBFT algorithm as the basis for reaching consensus. The consensus algorithm is meant to tolerate malicious nodes in the blockchain.

In [49], two forms of permissioned blockchains were proposed. A private blockchain is used to ensure proper authentication of users within the domain of IoT devices. Each such private blockchain is required to set up a special server called the blockchain proxy server and another server that is referred to as the key management center. Each private blockchain is in charge of maintaining the data in its domain. The paper stated that the data would be encrypted. These blockchain proxy servers from all domains form a consortium blockchain, the purpose of which is to facilitate cross-domain data sharing.

In [50], three collaborating custom blockchains were proposed to support secure operations of SAGIN—one blockchain per segment (i.e., ground, aerial, and space). Smart contracts were proposed to facilitate cross-chain operations, which the authors have claimed necessary to support SAGIN operations. The authors proposed the use of PBFT as the basis for consensus. For the space blockchain, some more trusted satellites are tasked with creating and verifying blocks. The aerial blockchain is run on trusted aircraft and ground stations. The ground blockchain runs on the devices in the ground network.

In [43], two custom blockchains were proposed using a non-mainstream consensus algorithm called the Conflux consensus protocol. The Conflux consensus protocol is based on a directed acyclic graph, and it boasts a throughput of over 100,000 transactions per second.

In [44], a custom private blockchain called hashchain was proposed. The blockchain nodes serve as security managers that are in charge of maintaining cross-border vehicle identity information. The custom blockchain relies on a distributed streaming platform called Apache Kafka to synchronize the identity records rather than a decentralized consensus algorithm. The purpose of the custom blockchain design is to offer low latency and high throughput.

The purpose of the custom private blockchain proposed in [47] is to support content fetching via a smart contract, with the blockchain nodes run on the UAVs. Some mobile user equipment requests specific content and pays a fee for the content, and the content provider sends the required contents to the user equipment, all via a smart contract. The content and the requests are packed into transactions, and the transactions are aggregated into blocks. The block-proposing node earns a reward. Unfortunately, no concrete implementation details were provided by the authors.

In [46], very little technical details were provided for the proposed custom blockchain. Base stations and roadside units run as blockchain nodes, and a so-called proof-of-authority algorithm is adopted as the consensus algorithm.

## 6. RQ3: Are the Blockchain-Based Solutions Valid for the Intended Purposes and Technically Sound?

For security and dependability research, typically, the expectation is for the authors to outline system models with the specific set of considered attack vectors, present at least an informal proof of correctness of the proposed solutions to mitigate the attack vectors, and often include experimental validation with a prototype implementation of the proposed solution in actual use cases [17,60]. Unfortunately, as shown in Table 5, the studies that we surveyed barely followed this scientific rigorousness. One study presented reasonably comprehensive security analysis of the proposed solution but without experimental validation [37]. Three studies claimed to have conducted experiments with an actual blockchain [43,45,49] (all used some forms of Hyperledger, which is an open-source framework for permissioned blockchains [58]). All three studies ran a very small set of blockchain nodes in a single physical computer with virtual machines. Most other studies used simulation for validation, and the focus was performance evaluation for normal operations, i.e., when there are no faults and no cyberattacks. One study included no form of validation at all [48]. Another study provided a general discussion on the roles that could be played by blockchain, and naturally included no form of validation [38]. In the following, we analyze each of the studies.

In [38], a smart contract is proposed to enforce the spectrum allocation scheme among the secondary users of the spectrum. This is consistent with the intention of the smart contract. Hence, the proposal is technically sound and could serve the proposed purpose. The study would have been more valuable if a concrete smart contract is provided with experimental evaluation. The study resorted to the use of simulation to evaluate the proposed solution.

One issue with the blockchain-based solution proposed in [37] lies in its custom lightweight consensus algorithm. First, no proof-of-correctness for the consensus algorithm is provided. Second, even if the algorithm is correct, it is apparent that the algorithm follows traditional distributed consensus design, which assumes a known and stable membership. As such, the algorithm is not decentralized, and the system depends on the algorithm is not a decentralized system. The fact alone contradicts the claims of the set of unique characteristics of the blockchain technology because the set of characteristics can only be achieved in a decentralized system (with a decentralized consensus algorithm) [31,53]. Yet, another issue with the study is that the proposed solution is not implemented. Without an actual implementation of the blockchain-based solution, the validity of the reported simulation results becomes questionable.

In [41], the blockchain is proposed to process and log the task processing operations as transactions. By itself, there is nothing wrong. However, the study claimed to use the PBFT algorithm for consensus. This means that the proposed blockchain does not ensure decentralized computing because PBFT requires a predefined membership. As such, the system would not offer the set of unique characteristics such as data immutability. While a correctly implemented system would still offer security similar to that of a traditional system, the PBFT algorithm as presented in the paper includes only the sub-algorithm for normal operation, i.e., only when there is no fault and there is sufficient synchrony in the system [17,59]. A view change algorithm is needed to ensure consensus in the presence of primary failure and strong synchrony [59]. Furthermore, in the presence of strong asynchrony, the PBFT might not terminate due to the Fisher-Lynch-Patterson (FLP) impossibility result [61], which means that the system would not make any progress (i.e., it would lead to a system throughput of 0 transactions per second). Besides this critical issue, the study failed to elaborate implementation details of the proposed blockchain. For example, by default, transactions in a blockchain are used to record transfer of ownership of some token used in the system. How to record the task processing information in the transaction, and how a blockchain node would verify the transactions and the blocks are not clear. Furthermore, the practicality of the proposed solution is questionable. First, running blockchain nodes on UAVs is problematic. Even if somehow a highly secure and efficient consensus algorithm is used, the blocks would grow indefinitely. It is unclear if it is practical to equip UAVs with huge persistent storage capability. Second, due to the high cost of launching the satellites into space, it is unclear if it is practical to offload the computational tasks to the satellites. The study presented in [40] share exactly the same technical problem where only the normal operation of the original PBFT is considered.

The blockchain architecture proposed in [39] for spectrum management itself appears to be technical sound. However, the details disclosed in the study revealed several issues. First, while the authors stated that the local blockchains and the regional blockchains are permissionless blockchains (which means any node would decide to join or leave on its own), the nodes in the local and regional blockchains are actually assigned according to the multi-chain dividing and updating algorithm. This means that the local and the regional blockchains cannot possibly be permissionless blockchains. Second, according to the blockchain spectrum trading mechanism presented, the regional blockchain is treated like a traditional distributed server, which can be invoked for arbitrary functions. While this issue can be resolved by using one or more smart contracts, the description demonstrated inadequate understanding of the blockchain technology. Another issue is common to most solutions that we reviewed in this paper, i.e., blockchain nodes must be nodes that are part of SAGIN. In fact, this is completely unnecessary. Blockchains support very lightweight computing devices as they’re users that issue transactions, as long as they are equipped with or have access to digital wallets. More elaboration will be provided in Section 7.

It is apparent that the blockchain proposed in [42] is not actually implemented. The proposed solution is validated using simulation and no technical details are provided in the paper. Nevertheless, the Tangle consensus algorithm is not fully implemented in IOTA, despite that the algorithm has been well publicized [56]. The current IOTA uses a centralized and trusted coordinator to synchronize the state across the blockchain nodes.

In [49], the stated primary purpose of using the blockchain technology is to ensure data immutability. Yet, private blockchains and a consortium blockchain are proposed. As we elaborated in [31,53], such permissioned blockchains are essentially centrally controlled, and have no intrinsic means to ensure data immutability. besides this serious issue, some claims made in the paper are technically problematic. For example, the authors claimed that the blockchain offers “unique data encryption and verification mechanism…” ([49], page 392). In fact, the blockchain technology does not offer encryption functionality. Instead, blockchain uses secure hashing and public-key digital signatures as the foundation for data and asset protection and token ownership verification.

In [50], the cited reason for proposing a blockchain-based solution is that blockchain would offer decentralization, stronger security, and smart contract. However, the proposed solution has several issues. First, the study failed to motivate why a separate blockchain is necessary for each segment of the network. Second, a blockchain would store all transactions, and as such, it is highly questionable to run a blockchain node on a satellite or an aircraft. Third, the choice of using PBFT as the consensus algorithm means the proposed blockchains are not decentralized systems after all. Unlike decentralized consensus algorithms such as proof-of-work, PBFT requires a predefined membership, which means that the systems are not open and not decentralized. Fourth, the assumption of using some trusted nodes for block creation and verification means the actual systems are centrally controlled, which is directly against the purpose of the blockchain technology. The proposed solution is validated with a home-grown blockchain simulator instead of actual experimentation.

The Conflux consensus protocol used in [43] to develop custom blockchains is not mainstream and it has not been rigorously scrutinized academically. The claim for offering over 100,000 transactions per second implies the consensus is achieved probably by centralized control. This paper claims to have based on the Hyperledger Fabric. However, the experimentation was done on a single Core i5 computer, which implies that a single blockchain node was used.

Rather similar to the approach taken in [43], another study [44] also proposed to use a non-mainstream custom blockchain. In the paper [44], the authors claimed the the blockchain would offer a distributed and decentralized solution to vehicle identity authentication. The combination of two terms “distributed” and “decentralized” is quite odd because a decentralized system for sure would be a distributed system, but not vice versa. What is proposed by the authors is apparently a distributed system with central control instead of a decentralized blockchain system. The fact that a streaming service is used instead of a consensus algorithm means that the custom “blockchain” cannot guarantee that a new block would consist of the same set of records at all blockchain nodes. In the journal version of the study [45], more simulation results are reported. The processing latency of the proposed hashchain with the streaming service is compared with that of PBFT. The paper did not disclose details on how the PBFT algorithm is implemented. Furthermore, it is clear that the authors did not consider the performance of the proposed solution in the presence of faults and cyber attacks.

In [48], the stated benefits of using blockchain for maritime communication were enhanced security and privacy, efficient authentication, and automation with smart contracts. While blockchain technology could potentially enhance the security and privacy if used properly and smart contracts are very attractive features to enable secure and fault-tolerant automation of operations, a blockchain alone does not facilitate proper authentication. This is because. as a decentralized system, a public blockchain is open to anyone to join with a pair of private–public keys. This design is intended to offer a degree of anonymity to its users (for privacy protection of its users). As such, the design is vulnerable to Sybil attacks, and additional mechanisms are needed to mitigate the issue [25].

In [47], the stated benefits of using blockchain for content delivery include that the blockchain ensures data immutability (i.e., tamper-proof), among others. The proposed blockchain is supposed to run among the UAVs. As we have argued in other publications [31,53], the data immutability of an open system can only be achieved with a high cost of achieving consensus, which is reflected in the copy of the chain of blocks maintained by all blockchain full nodes. It is the high cost of altering the chain that serves as the barrier to modification of the data recorded in the blockchain. It is unlikely for a blockchain run on a group of UAVs to accomplish this objective.

In [46], the choice to use proof of authority as the consensus algorithm conflicts with the goal of achieving a decentralized, trustworthy operating environment because proof of authority is essentially a centralized decision-making algorithm.

In summary, virtually all studies suffer from some form of technical issues. Although all studies aimed to use blockchain technology to create a trusted operating environment for SAGIN operations or applications, the use of custom private blockchains means that the proposed solutions are, in fact, invalid (one study mentioned that two of the blockchains they proposed can be public blockchains, but based on the context, the blockchains are inevitably permissioned [39]). That said, based on the context of the studies, decentralization and data immutability are not necessarily the most essential objectives. Quite often, what is needed is a distributed, dependable system with a specific set of functionalities. In this case, a custom private blockchain would be a good fit, provided that a sound distributed consensus algorithm is used.

## 7. Discussion

Ideally, a comprehensive review should provide some quantitative meta analysis so that some new knowledge and insight can be drawn from the reviewed studies. Unfortunately, because very few studies in our review provided experimental results (most only validated their proposed solutions via simulation), it is not practical for us to perform such quantitative analysis. Nevertheless, in this section, we propose a guideline for developing blockchain-facilitated SAGIN solutions and report our findings resulting from our examination of research on security and SAGIN for future research opportunities.

### 7.1. Guideline for Blockchain-Facilitated SAGIN Solutions

At the beginning of this study, we had an additional research question regarding the guideline proposed for using blockchain in SAGIN operations and applications because similar guidelines have been proposed in other disciplines, such as smart grids [32]. Unfortunately, we found no study that provides such a guideline. The closest is a “tailored blockchain” for SAGIN with IoT proposed in the only review paper on the topic of blockchain and the IoT in SAGIN [3]. This “tailored blockchain” can be considered a summary of the custom blockchains proposed in various studies for SAGIN, and it can also be considered a blueprint for future custom blockchains for SAGIN.

The “tailored blockchain” consists of six layers, and it appears to have originated in IoT research [62,63,64]. In the following, we introduce and elaborate on the “tailored blockchain”, as illustrated in Figure 5 of [3], from bottom up, as follows:

- Data layer: This layer defines how the data are recorded in the blockchain, including a “redesigned block structure” (presumably referring to the customization of the block structure for SAGIN and IoT data), an “editable blockchain” (this is not elaborated upon in [3]), DAG (short for directed acyclic graph, which refers to the data structure introduced in IOTA [56] where the transaction bundles are chained together as a graph), and off-chain (this is odd because off-chain is in contrast to the data placed on the main blockchain; if the data are to be placed off the main chain, then the data may be stored in many different forms, such as files in the InterPlanetary File System [65]).
- Network layer: “Satellite and UAV communications” (this may be needed to connect to blockchain users but should not be used for blockchain full nodes, as elaborate upon later), sharding (this refers to a scaling technique that partitions the blockchain network into several parts for increased throughput [66]), SDN (short for software-defined networking [67]), and NFV (short for network function virtualization [68]).
- Consensus layer: IoT-specific consensus protocols (indeed, several studies that we reviewed proposed lightweight algorithms for higher throughput).
- Incentive layer: “Well-designed incentives” (presumably, the incentive scheme could be designed specifically for SAGIN operation and applications).
- Contract layer: “AI-driven secure contracts” (the paper did not elaborate on what it means by AI-driven).
- Business layer: “multiple blockchains and sidechains” (it is odd to include this issue as part of the business layer), “cross-chain mechanism” (it could be implemented via smart contracts), and regulated blockchain (the paper did not elaborate, but it could mean that the blockchain design should incorporate mechanisms for meeting government regulations).

Next, we comment on this design. First and foremost, we emphasize the principle of not reinventing the wheel. Considering the maturity of Bitcoin, Ethereum, and Hyperledger and the availability of smart contracts, we see no reason to customize the internals of the blockchain, such as block and transaction data structures, or incentive schemes. Smart contracts allow users of the blockchain to design sophisticated data structures to store data for particular applications. Smart contracts also facilitate the creation of custom tokens, which support custom incentive schemes for participating in the blockchain. Second, the network protocols and higher-level algorithms (such as the consensus algorithm) in the blockchain run over the TCP/IP protocol stack, and they are ignorant to the low-level networking technologies, be they satellite communication, UAV communication, SDN, or NFV. Of course, a full-node operator may decide to connect to the Internet via a specific low-level networking technology. Third, we caution on the use of traditional distributed consensus algorithms such as PBFT or RAFT (together with Hyperledger, for example). While many publications have claimed superior throughput for these consensus algorithms, the reported superior numbers are only for normal operations when the operating environment is sufficiently synchronous [17]. Such algorithms may fail to make any progress at all when the operating environment is asynchronous or when the network is subject to cyber attacks (particularly under denial-of-service attacks) due to the FLP impossibility result [61]. Also due to the FLP impossibility result, an unreliable failure detector (often via timeout) must be used to determine if another node has failed in traditional consensus algorithms. The unreliability of the failure detector inevitably would lead many corner cases, which would make the implementation of these traditional consensus algorithms highly complex, error-prone, and brittle.

To accommodate the need for higher throughput and lower transaction fees, numerous scaling solutions, including the use of multiple blockchains, side chains, and layer-2 blockchains with cross-chain mechanisms, have been introduced by the blockchain community [69]. That said, positioning these issues as part of the business layer is confusing because they appear to be part of the blockchain architecture.

Furthermore, unlike some arbitrary piece of code, a smart contract must run deterministically and passively, i.e., given the same input, the contract would generate the same output and make the same state transitions, and the contract cannot run on its own (such as via a thread with a timer). As such, it is unclear what it meant by AI-driven secure contract. While AI can help decide when to invoke a smart contract, a smart contract cannot internally make any AI-driven statistical predication because it would lead to nondeterminism [70].

The “tailored blockchain” outlined in [3] may be of some value to developers that have a very good reason to create a custom private or consortium blockchain for SAGIN, provided that they are aware of the issues that we have identified above. However, we argue that in most cases, a custom permissioned blockchain is unnecessary, and it may be cost-prohibitive, considering that one would have to maintain the blockchain full nodes. We strongly encourage the SAGIN community to use existing large public blockchains such as Bitcoin and Ethereum and, when necessary, use existing layer-2 blockchain platforms such as Polygon [71]. Perhaps more importantly, the SAGIN community may find a guideline useful regarding whether blockchain is a good option for solving the problems they face and, if so, what kind of blockchain should be used.

We construct the guideline based on both functional and non-functional requirements of SAGIN operations and applications. We note that the functional and non-functional requirements are orthogonal, and as such, they can be considered separately. Functional requirements include our findings for research question 1, such as dynamic spectrum management, mobility management, vehicle ad hoc network applications, IoT applications, and global content delivery. The non-functional requirements focus on the set of unique characteristics of the blockchain technology, including data immutability, data provenance, censorship resistance, transparency, and decentralization. We intentionally omit non-functional requirements for security and trust because one could argue they can be accomplished by all forms of blockchain and traditional systems.

**Figure 5:**
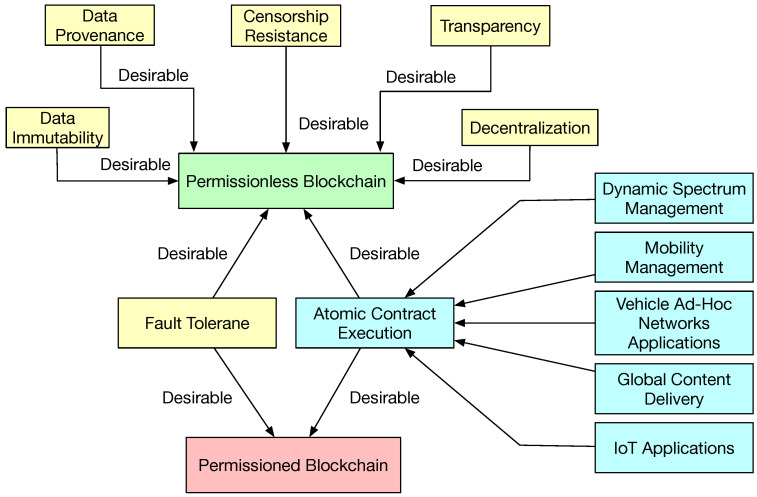
Guideline on blockchain adoption in SAGIN.

As shown in Figure 5, according to our guideline, virtuallyall SAGIN operations and applications can be implemented via smart contracts. If one or more of the unique set of blockchain characteristics are requirements, then permissionless, i.e., public, blockchains should be used. If, on the other hand, one just needs a fault-tolerant and secure distributed system (with smart contract support), then permissioned blockchains (private or consortium blockchains) may be used. Only when a permissioned blockchain is desirable is a custom private or consortium blockchain necessary.

**Figure 6:**
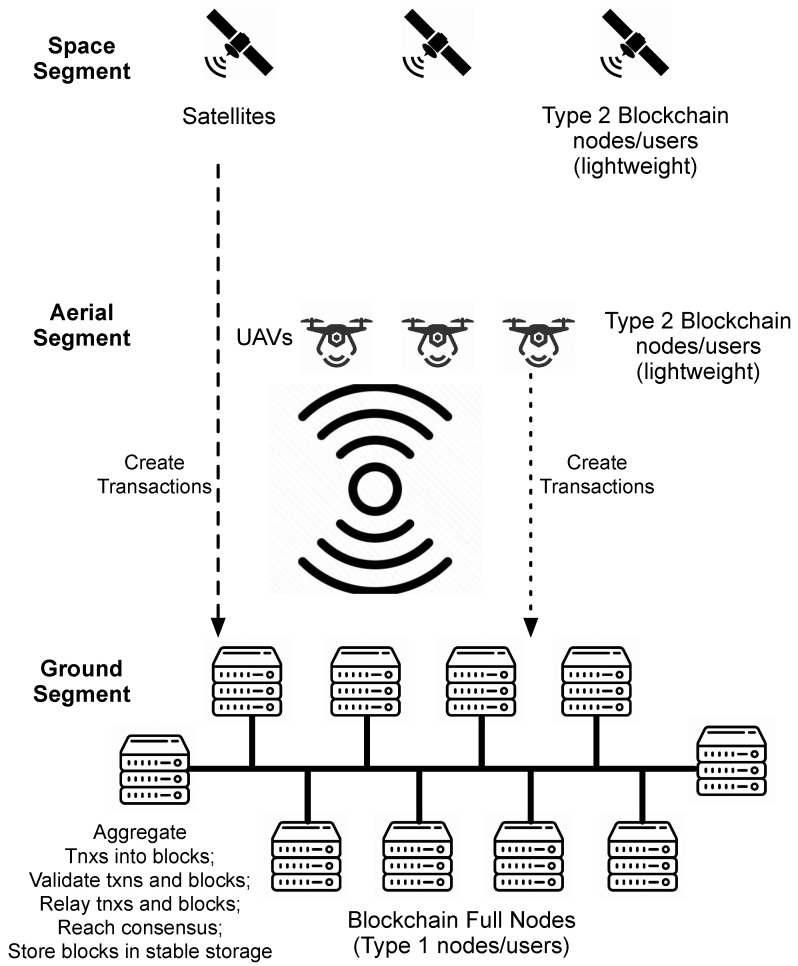
Blockchain full nodes should be deployed on servers in the ground segment.

If a permissioned blockchain is desirable, the next step is to decide on where to deploy the blockchain full nodes, which are responsible for supporting the core operations of the blockchain. Several studies have claimed to deploy the blockchain in the space segment among satellites or in the aerial segment among UAVs, without explicit elaboration on why such a decision was made. Presumably, this is to reduce the delays in transmission and propagation between different blockchain full nodes. However, this design ignores the limited computing and storage capability of satellites and UAVs. Furthermore, transferring the potentially large amount of data maintained by the blockchain would cause significant delays in transmission. It is a much better choice to deploy the blockchain full nodes in workstation/servers connected via high-bandwidth cables (such as fiber optics) in the ground segment, as shown in Figure 6.

Note that a blockchain has two different types of users (as well as nodes): (1) those that run blockchain full nodes, which are in charge of aggregating transactions into blocks, validating the transactions and blocks, solving consensus puzzles, relaying the transactions and blocks, and storing the chained blocks in stable storage, and (2) those that join the blockchain with a digital wallet as a lightweight node, which do not participate in the core blockchain operations. The second type of users may come and go as needed, while the first type of users are supposed to operate all the time, non-stop. Furthermore, the first type of user maintains the blockchain system, including the collection and safeguarding of transactions, and the second type of user generates transactions (with the exception of the coinbase transaction, which is the first transaction included in each block and is created by the block creator to claim the block rewards and the transaction fees). As such, the delay between the second type of user and the blockchain is not critical to the operation of the blockchain. However, the delay between the first type of node is mission-critical to the integrity, safety, and security of the blockchain. As can be seen in Table 2, except for GEO and MEO satellites, the one-way propagation delay to the ground is negligible (<10 ms). The propagation delay for UAVs to the ground stations is even less (<0.1 ms). This further demonstrates that there is no reason to deploy blockchain full nodes in UAVs or LEOs.

### 7.2. Opportunities for Blockchain Research in SAGIN

Here, we report the findings regarding whether there are opportunities in SAGIN for blockchain to be used to enhance security by examining the entries returned with the search term “SAGIN and security”. As shown in Figure 1, 11 of 44 entries overlap between the search term “SAGIN and blockchain” and the search term “SAGIN and security”, which we already examined in depth. Among the 33 entries, 10 are obviously not focused on security, so were excluded from our study. Ten of the remaining studies focused on issues in which blockchain technology could play a role, while blockchain technology would hardly be useful for the remaining 13 studies, as summarized in Table 6. As can be seen, there are opportunities to incorporate blockchain technology in designing solutions for secure routing [72,73] and some other applications, such as UAV tracking [74].

Finally, although not cited as a reason for using blockchain technology in the studies that we reviewed, interoperability could be facilitated by blockchain as a platform for trusted data sharing and coordination of operations [22]. This could also be an opportunity for future development in SAGIN.

## 8. Conclusions

In this article, we present a systematic review on blockchain-enabled solutions for SAGIN operations and applications. This review is guided by three research questions: (1) what SAGIN operations (and applications) have been enhanced by blockchain technology, (2) how blockchain technology is used in the proposed solutions, and (3) whether the blockchain-based solutions valid for the intended purposes and technically sound.

The findings for the first research question show that blockchain technology has been proposed to enhance the security of core SAGIN operations, specifically for dynamic spectrum management and mobility management. With the help of mobile edge computing, some studies expanded the notionof spectrum sharing to resource sharing, where resources could be the spectrum (bandwidth), energy, time, or computation [41,42].

The findings for the second research question reveal that custom private or consortium blockchains with non-mainstream consensus algorithms are the predominant approach to blockchain-based solutions for SAGIN. This is rather odd based on our observations on the application of blockchain in other disciplines, such as smart grids [32], where smart contracts are heavily used.

The findings for the third research question uncover serious issues in the proposed blockchain-based solutions. Quite often, the rationale for using a blockchain-based solution is not well-justified. Decentralization and data immutability are the most commonly cited reasons for using blockchain technology. However, private or consortium blockchains have been proposed, which are not decentralized, nor do they ensure data immutability [31,53]. Very few studies have carried out experiments with an actual blockchain system, and the great majority of studies resorted to using simulation to highlight the benefits of using the proposed blockchain-based solution. Furthermore, some proposed non-mainstream consensus algorithms suffer from technical mistakes (such as only considering the normal operation of the system when there is no fault and no strong asynchrony) [17].

To help address the issues that we identified, we developed a guideline on using blockchain in SAGIN. The guideline considers two sets of user requirements. One set is about the non-functional (i.e., quality-of-service) requirements, which are defined by the set of unique characteristics offered by blockchain technology. The other set is about the functional requirements for SAGIN operations and applications. We hope the guideline helps developers in SAGIN make the right decision as to whether or not to adopt blockchain technology and how to construct a blockchain-based solution if the use of a blockchain is desirable. Finally, we examined the research on security in SAGIN and identified a few research directions in which blockchain technology could play a role.

## Figures and Tables

**Table 1:** Literature collection results.

Search Term	Total No. of Pubs	Year	No. of Pubs
SAGIN	261	2024	64
		2023	69
		2022	67
		2021	29
		2020	18
		2019	12
		2018	2
SAGIN & IoT	55	2024	14
		2023	16
		2022	15
		2021	7
		2020	1
SAGIN & Security	44	2024	8
		2023	16
		2022	12
		2021	3
		2020	4
		2019	1
SAGIN & Blockchain	17	2024	2
		2024	6
		2022	5
		2020	3
		2019	1
SAGIN & IoT & Security	11	2024	2
		2023	3
		2022	5
		2021	1
SAGIN & Blockchain & Security	11	2024	1
		2023	2
		2022	5
		2020	2
		2019	1
SAGIN & Blockchain & IoT	6	2024	2
		2023	2
		2022	2
SAGIN & Blockchain & IoT & Security	5	2024	1
		2023	2
		2022	2

**Table 2:** Key characteristics of segments in SAGIN.

Segment	Implementation	Propagation Distance	Propagation Delay
Space	GEO	35,786 km	∼120 ms
	MEO	2000 km–35,786 km	[∼6.7 ms, ∼120 ms]
	LEO	160 km–2000 km	[∼0.53 ms, ∼6.7 ms]
Aerial	HAP	17–22 km	[∼0.057 ms, ∼0.073 ms]
	LAP	<10 km	<0.033 ms
Ground	Cellular/WiMaX/WiFi	<40,075 km	<133.6 ms

**Table 4:** SAGIN How blockchain is used in SAGIN operations and applications.

Blockchain-Based Solution	Consensus Algorithm	Blockchain Full Nodes	Reference(s)
A custom private blockchain for incentive-based auction of spectrum	Delegated proof of stake	UAVs	[37]
A custom private blockchain	PBFT	UAVs	[41]
A smart contract (no details)	N.A.	N.A.	[38]
Local blockchain (for spectrum sharing between the primary users and the secondary users), regional blockchain (for spectrum trading and interference control), and global blockchain (for data synchronization and cross-chain transactions)	N.A.	N.A. (local and regional blockchains are public; global blockchain is a consortium blockchain)	[39]
Two custom private blockchains	Adapted PBFT	Ground base stations	[40]
A custom private blockchain that supports smart contracts	Directed acyclic graph (Tangle)	Symbiotic radios (ground base stations and UAVs)	[42]
A private blockchain for user authentication in each domain and a consortium blockchain for cross-domain data sharing	RAFT	For the private blockchain, only pre-selected nodes are allowed to create blocks; for the consortium blockchain, the blockchain proxy servers consist of the blockchain nodes	[49]
A private blockchain that supports smart contracts	N.A.	UAVs	[47]
A custom private blockchain for vehicle authentication	Uses a distributed streaming platform instead of consensus	N.A	[44,45]
A custom private blockchain for vehicle authentication	Proof of authority	N.A.	[46]
Three collaborating custom blockchains, (one per segment). Smart contracts are used to facilitate cross-chain operations.	PBFT as the basis	Devices in each segment	[50]

**Table 5:** Summary of the validation methods in the proposed blockchain-based solutions and our comment.

Implementation	Validation	Comment	References
Hyperledger Caliper	Experiment with 1 ordering node, 3 peer nodes, and 2 KMC nodes running in VMware	The use of permissioned blockchains contradicts to the goal of ensuring data immutability	[49]
Hyperledger Fabric	Experimented with single Windows Core-i5 Computer	It is hardly believable for any system to attain 100,000 transactions per second!	[43]
No evidence	Simulation with SUMO, OM-Net++, and Veins	No proof is presented for using a streaming server instead of a sound consensus algorithm can ensure the correctness of the proposed blockchain	[44]
Hyperledger Fabric (claimed)	Simulation with SUMO, OM-Net++, and Veins, and experiment run on a single Alibaba cloud server	Blockchain-related experiment is done for assessing delays in generating blocks and in block authentication without considering the complex scenarios an actual blockchain would encounter	[45]
No evidence	Security analysis and simulation	The use of a so-called lightweight consensus algorithm contracts with the goal of achieving the unique set of properties of the blockchain technology. That said, the solution proposed actual fits the stated objectives of mitigating malicious spectrum bidders and avoiding a single point of failure	[37]
No evidence	Simulation with NS3	No details provided for the smart contract	[38]
No evidence	Simulation	The proposed spectrum trading functionality of the regional blockchain can only be accomplished via smart contracts instead of via basic transactions. Blockchain nodes do not have to be part of SAGIN.	[39]
No evidence	Simulation	The use of PBFT to reach consensus means that the solution is not decentralized and cannot ensure data immutability	[40]
No evidence	Simulation	The use of PBFT to reach consensus means that the solution is not decentralized and cannot ensure data immutability	[41]
No evidence	Simulation	The Tangle algorithm is not yet robust against double-spent attacks, and a centralized trusted coordinator is relied on by IOTA	[42]
No evidence	Simulation	It is unlikely for a blockchain deployed on UAVs to ensure data immutability	[47]
No evidence	Simulation	The use of a centralized consensus algorithm (proof-of-authority) contracts to the stated goal of establishing decentralized trustworthy operating environment	[46]
No evidence	Simulation (developed with Go language)	The use of three separate blockchains (one per segment of SAGIN) is not justified. The use of PBFT for consensus contracts the goal of decentralization. No details for the smart contract is disclosed.	[50]
No evidence	None	The claim of using blockchain to facilitate authentication is problematic because anyone could create a pair of keys and join as a user, which is vulnerable to Sybil attacks	[48]

**Table 6:** Studies on security in SAGIN with respect to the potential of blockchain application.

Applicability of Blockchain	Security Research in SAGIN	Reference(s)
Blockchain could play a role	Secure handover, which is an essential step in mobility management	[75]
	Secure task scheduling, which is related to resource sharing and service exchange	[76]
	Resource scheduling, which is related to resource sharing and service exchange	[77]
	Physical unclonable function (PUF)-based authentication and key distribution; blockchain could help, provided that a user/device registration step is implemented	[78]
	Data sharing, which can be facilitated via smart contracts	[79]
	Secure routing	[72,73]
	UAV tracking	[74]
	Security reference architecture proposed for SAGIN-powered smart cities	[80]
	Trust management in emergency message dissemination in SAGIN	[81]
Not appropriate for blockchain	Physical layer security	[82,83,84,85,86,87,88]
	Quantum key distribution in resource allocation	[89]
	Data encryption scheme (multi-authority ciphertext policy attribute-based encryption with dynamic revocation)	[90]
	Encryption decision method	[91]
	Quantum-proof security	[92,93]
	PUF-based key agreement	[94]

## Data Availability

Not applicable.

## Abbreviations

The following abbreviations are used in this manuscript:

SAGIN	Space–Air–Ground Integrated Network
IoT	Internet of Things
GEO	Geostationary
MEO	Medium Earth Orbit
LEO	Low Earth Orbit
UAV	Unmanned Aerial Vehicle
HAP	High-Altitude Platform
LAP	Low-Altitude Platform
PBFT	Practical Byzantine Fault Tolerance
MEC	Mobile (also Multiacces) Edge Computing
FLP	Fisher–Lynch–Patterson
SDN	Software-Defined Networking
NFV	Network Function Virtualization
TCP	Transmission Control Protocol
IP	Internet Protocol
DAG	Directed Acyclic Graph
